# Bone mineral density in women newly diagnosed with breast cancer: a prospective cohort study

**DOI:** 10.1038/s41523-022-00388-z

**Published:** 2022-02-17

**Authors:** Merav Fraenkel, Victor Novack, Yuval Mizrakli, Michael Koretz, Ethel Siris, Larry Norton, Tali Shafat, David B. Geffen

**Affiliations:** 1 Endocrine Unit, Soroka University Medical Center and the Faculty of Health Sciences, Ben-Gurion University of the Negev, Beer Sheva, Israel; 2 Clinical Research Center, Soroka University Medical Center and the Faculty of Health Sciences, Ben-Gurion University of the Negev, Beer Sheva, Israel; 3 Breast Health Center, Soroka University Medical Center and the Faculty of Health Sciences, Ben-Gurion University of the Negev, Beer Sheva, Israel; 4 Division of Endocrinology, Columbia University Medical Center, New York, USA; 5 Breast Center, Memorial Sloan Kettering Cancer Center, New York, USA; 6 Department of Oncology, Soroka University Medical Center and the Faculty of Health Sciences, Ben-Gurion University of the Negev, Beer Sheva, Israel

**Keywords:** Breast cancer, Epidemiology

## Abstract

Estrogen may have opposing effects on health, namely increasing the risk of breast cancer and improving bone health by increasing bone mineral density (BMD). The objective of this study was to compare dual-energy X-ray absorptiometry (DXA) BMD between women newly diagnosed with breast cancer and matched controls without breast cancer. Women newly diagnosed with breast cancer treated between April 2012 and October 2017 were prospectively enrolled. A control group was established of women with negative mammography or breast ultrasound, matched 1:1 by age, body mass index, parity, and the use of hormone replacement therapy. All those included had DXA BMD, and lab assessments at enrollment. Of 869 women with newly diagnosed breast cancer, 464 signed informed consent. Of the 344 who completed the study protocol, 284 were matched to controls. Overall, the mean age was 58 years. Compared to the control group, for the breast cancer group, the mean vitamin D level was lower (48.9 ± 19.0 vs. 53.8 ± 28.8 nmol/L, *p* = 0.022); and mean values were higher of total hip BMD (0.95 ± 0.14 vs. 0.92 ± 0.12 g/cm^2^, *p* = 0.002), *T* score (−0.38 ± 1.17 vs. −0.68 ± 0.98, *p* = 0.002), and *Z* score (0.32 ± 1.09 vs. 0.01 ± 0.88, *p* < 0.001). Among the women with breast cancer, no correlations were found of baseline BMD with tumor size or grade, nodal involvement, or breast cancer stage. We concluded that women with newly diagnosed breast cancer tend to have higher BMD than women with similar characteristics but without breast cancer. This implies that BMD might be considered a biomarker for breast cancer risk.

## Introduction

The incidences of both breast cancer and osteoporosis increase following menopause^1^. Estrogen is central for the maintenance of bone integrity; its deficiency leads to accelerated bone loss and decreased bone mineral density (BMD), and a propensity for osteoporotic fractures^2^. BMD has been suggested as a marker for lifetime estrogen exposure, and thus may aid in predicting breast cancer risk^3^. High endogenous estrogen levels have been shown to be associated with increased risk of breast cancer (particularly hormone receptor-positive breast cancer)^4^. Reducing estrogen levels (e.g., with the administration of aromatase inhibitors) has been shown to reduce the risk of breast cancer recurrence^5^.

Several observational studies have shown an association of higher BMD with increased breast cancer risk irrespective of other risk factors^2,6–16^. In a retrospective study of over 14,000 women who performed dual-energy X-ray absorptiometry (DXA) BMD, we found that future breast cancer risk adjusted for age and body mass index (BMI)) was doubled in women in the highest tertile compared to the lower tertiles of femur neck and total hip BMD *Z* scores^17^. In contrast, other studies found no association between BMD and breast cancer risk in either pre or postmenopausal women^18–23^. In a meta-analysis published during the past decade, which included over 70,000 postmenopausal women, high hip and spine BMD was found to be a risk factor for developing breast cancer. This supports the conclusion that higher BMD confers higher breast cancer risk^15^.

In addition to estrogen exposure, several other factors, including vitamin D levels, may influence BMD and breast cancer risk. Low vitamin D levels may lead to reduced bone mineralization and therefore lower BMD^24^. Findings on the association of 25 OH-vitamin D levels and the risk of breast cancer are conflicting^25,26^. A review by Bauer et al., including nine prospective studies with a total of 11,656 women, found that postmenopausal (but not premenopausal) breast cancer risk was decreased by 12% for each 5 ng/mL increase in 25(OH)D level between 27 and 35 ng/ml^27^. Similar observations of an inverse “dose–response” relation between 25(OH)D levels and breast cancer risk were also found in additional meta-analyses^28,29^.

We conducted a prospective matched cohort study with the primary objective of assessing BMD in women with newly diagnosed breast cancer, and comparing to matched controls.

## Results

### Study population

**Fig. 1:**
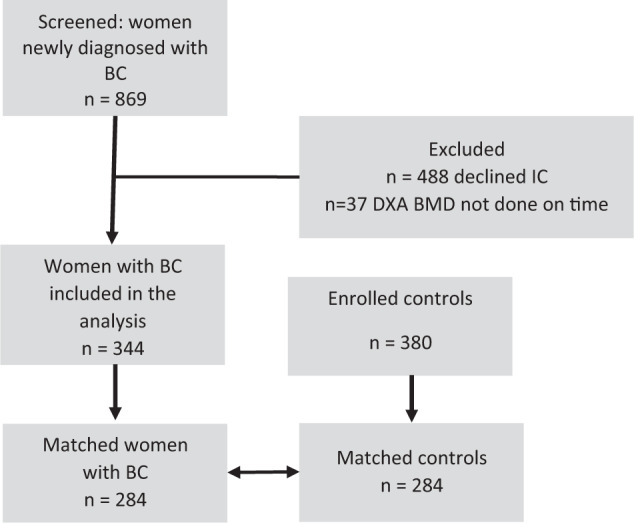
Flow chart of study population.

Between April 2012 and March 2016, 869 women newly diagnosed with breast cancer were referred to the oncology department of our institute. Figure 1 shows the study population flow chart. Of the 869 patients, 381 gave their consent to participate in the study. Thirty-seven women were excluded from the analysis because the first BMD was not done within three months of enrollment, the predesignated time frame. Between April 2012 and October of 2017, 380 women were enrolled to the control group. In the final analysis 344 women with breast cancer (cases) were included; matching to controls was possible for only 284 of them.

Baseline characteristics of the women in the case and matched control groups are presented in Table 1. Mean age (58.8 ± 10.5 vs. 58.6 ± 9.5; *p* = 0.834), mean BMI (28.1 ± 5.0 vs. 27.6 ± 4.6; *p* = 0.201), and the use of hormone replacement therapy (HRT) (27.5%) did not differ statistically between the groups; this was expected, since these were the parameters used for matching. Although the participants were matched by parity, this parameter was lower in the breast cancer than control group (mean 2.8 ± 1.4 vs. 3.0 ± 1.1, *p* = 0.038). Ninety-seven percent of those with breast cancer and 100% of the control group were of Jewish origin. Rates of prior use of anti-osteoporotic medications in the two years prior to enrollment were collected from electronic medical records only for women insured by Clalit Health Services (70% of the participants in the breast cancer group and 97% of those in the control group), and were similarly low in the two groups. Considering only those with available blood tests, mean creatinine, eGFR, and parathyroid hormone (PTH) levels did not differ statistically between the two groups. Compared to the control group, for the breast cancer group, mean 25-OH vitamin D levels were lower (48.6 ± 18.8 vs. 53.8 ± 29.0; *p* = 0.017); and a higher proportion had a vitamin D level below 50 nmol/L (54.4% and 46.3%) (*p* = 0.071).

**Table 1:** Baseline characteristics of the breast cancer and control groups.

Group	Breast cancer *n* = 284	Control *n* = 284	*p*-value
*Age*			*0.834*
Mean ± SD	58.8 ± 10.5	58.6 ± 9.5	
Median (Q1,Q3)	60.5 (51,72.5)	60 (53,65)	
Range	30–83	26–79	
BMI mean ± SD	28.1 ± 5.0	27.6 ± 4.6	0.201
HRT use *n* (%)	78 (27.5%)	78 (27.5%)	1.0
Parity median (Q1, Q3)	3 (2,4)	3 (2,4)	0.038
Mean ± SD	2.8 ± 1.4	3.0 ± 1.1	
*Ethnicity* *n* (%)			*0.015*
Jewish	277 (97.5%)	284 (100.0%)	
Bedouin	7 (2.5%)	0 (0.0%)	
*Treatment for osteoporosis in the 2 years before enrollment*^a^			
Oral bisphosphonates *n* (%)	13 (6.6%)	15 (5.4%)	0.694
Injected bisphosphonates *n* (%)	2 (1.0%)	1 (0.4%)	0.380
Bisphosphonates (any) *n* (%)	15 (7.6%)	16 (5.8%)	0.456
Treatment with Vitamin D/Ca supplements *n* (%)	94 (47.5%)	145 (52.5%)	0.306
*Lab results at enrollment*			
Creatinine (mg/dL)			0.308
Mean ± SD	0.69 ± 0.16	0.70 ± 0.13	
*n* available data	281	277	
eGFR (MDRD calc; mL/min/1.73 m²) mean ± SD	98.7 ± 23.4	95.1 ± 20.3	0.053
PTH (pg/mL) mean ± SD	53.0 ± 28.8	55.2 ± 24.8	0.358
*n* available data	254	260	
(OH)25-Vitamin D (nmol/L) mean ± SD	48.9 ± 19.0	53.8 ± 28.8	0.022
*n* available data	261	268	
(OH)25-Vitamin D < 50(nmol/L) *n (%)*	142 (54.4%)	124 (46.3%)	0.068

*BMI* body mass index, *HRT* hormone replacement therapy, *eGFR* estimated glomerular filtration rate, *PTH* parathyroid hormone.

^a^Used drug distribution data for both sub groups, % of Clalit insurees is shown.

### BMD: case-control analysis

Results of DXA–BMD according to site, for the 284 women with breast cancer, and the 284 matched controls are presented in Table 2, expressed as both g/cm^2^ and as *T* and *Z* scores. Compared to the control group, for the breast cancer group, higher mean values were observed of femoral neck *Z* score (0.14 ± 1.02 vs. −0.02 ± 0.83; *p* = 0.042), total hip BMD (0.95 ± 0.14 vs. 0.92 ± 0.12; *p* = 0.002), *T* score (−0.38 ± 1.17 vs. −0.66 ± 0.98; *p* = 0.002), and *Z* score (0.32 ± 1.09 vs. 0.01 ± 0.88; *p* < 0.001). Mean values of the lumbar spine BMD, and *T* and *Z* scores did not differ statistically between the case and control groups (Table 2). Sub analysis according to menopausal status was not feasible since it was not documented for controls. Yet, our data showed that the mean menopausal age in the study population (BC group) was 49.4∓5.3 years. Splitting the whole study population by the age of 50 (presumed mean age at menopause), showed that femur neck and total hip BMD results were significantly higher in BC patients compared to controls only for those above age 50 while no differences were observed in the younger group. Statistical differences were not found between BC and controls in the percentage of participants fulfilling the WHO densitometric diagnosis of osteoporosis (*T* score < −2.5): at the femoral neck (12 (4.2%) vs. 4 (1.4%), *p* = 0.073); total hip (9 (3.2%) vs. 8 (2.8%), *p* = 1.0); and the lumber spine (33 (11.6%) vs. 25 (8.8%), *p* = 0.332).

**Table 2:** Bone mineral density for women with breast cancer and a matched control group.

	Breast cancer *n* = 284	Controls *n* = 284	*P*-value
*Femur neck*			
BMD (g/cm^2^)			0.133
Mean ± SD	0.89 ± 0.13	0.87 ± 0.11	
Median (Q1,Q3)	0.88 (0.79,0.97)	0.86 (0.79,0.93)	
Range	0.60–1.26	0.66–1.20	
*T-Score*			0.133
Mean ± SD	−0.78 ± 1.10	−0.91 ± 0.95	
Median (Q1,Q3)	−0.87 (−1.59,−0.10)	−0.99 (−1.59,−0.38)	
Range	−3.20 to 2.30	−2.71 to 1.84	
*Z-Score*			0.042
Mean ± SD	0.14 ± 1.02	−0.02 ± 0.83	
Median (Q1,Q3)	0.07 (−0.54,0.69)	−0.11 (−0.61,0.50)	
Range	−2.99 to 3.54	−2.19 to 2.54	
*T* score ≤ −2.5 *n* (%)	12 (4.2%)	4 (1.4%)	0.073
Total hip			
BMD (g/cm^2^)			0.002
Mean ± *SD*	0.95 ± 0.14	0.92 ± 0.12	
Median (Q1,Q3)	0.96 (0.85,1.05)	0.92 (0.83,0.99)	
Range	0.62–1.37	0.64–1.29	
*T*-Score			0.002
Mean ± SD	−0.38 ± 1.17	−0.66 ± 0.98	
Median (Q1,Q3)	−0.37 (−1.29,0.39)	−0.68 (−1.39,−0.11)	
Range	−3.14 to 3.05	−2.98 to 2.38	
*Z*-score			<0.001
Mean ± SD	0.32 ± 1.09	0.01 ± 0.88	
Median (Q1,Q3)	0.34 (−0.41,0.92)	−0.02 (−0.61,0.60)	
Range	−3.36 to 3.66	−2.69 to 2.67	
*T* score ≤ **−**2.5 *n (%)*	9 (3.2%)	8 (2.8%)	1.0
Spine (L1–L4)			
BMD (g/cm^2^)			0.171
Mean ± SD	1.11 ± 0.17	1.09 ± 0.16	
median (Q1,Q3)	1.08 (0.98,1.23)	1.06 (0.97,1.19)	
Range	0.73–1.64	0.70–1.61	
*T-Score*			0.171
Mean ± SD	−0.62 ± 1.40	−0.78 ± 1.34	
Median (Q1,Q3)	−0.81 (−1.70,0.41)	−0.98 (−1.76,0.07)	
Range	−3.73 to 3.87	−4.02 to 3.60	
*Z-Score*			0.085
Mean ± SD	0.25 ± 1.31	0.06 ± 1.22	
Median (Q1,Q3)	0.11 (−0.65,1.09)	−0.04 (−0.78,0.85)	
Range	−2.50 to 4.91	−2.60 to 4.49	
*T* score ≤ **−**2.5 *n* (%)	23 (8.1%)	20 (7.0%)	0.751
*T* score ≤ **−**2.5 on any site *n* (%)	33 (11.6%)	25 (8.8%)	0.332

*BMD* bone mineral density.

### Women with breast cancer

Baseline characteristics of women with breast cancer (including matched and unmatched participants, *n* = 344) are presented in Table 3. The mean age at breast cancer diagnosis was 58.0 ± 11.6 years and the mean BMI 28.6 ± 5.4 g/cm^2^. Most (67.2%) never smoked, 74.7% were postmenopausal, 27.7% used postmenopausal HRT, 24.4% reported having a previous fracture. The median number of pregnancies was three; 68% reported breastfeeding after every delivery. Fifty-three per cent were of Ashkenazi Jewish ancestry, 37% were Sephardic Jews, and 3% were Bedouin Arabs. The duration of unopposed lifetime estrogen exposure was calculated at 14.3 ± 4.4 years. The mean vitamin D level was 47.2 ± 19.2 nmol/L (normal range: 75–250 nmol/L), while the mean creatinine, eGFR, and PTH levels were within the normal range. Characteristics of breast cancer including: histologic type and grade, tumor-node-metastasis (TNM) stage, and immunohistochemistry (IHC) positivity for estrogen receptor (ER), progesterone receptor (PR), and human epidermal growth factor receptor-2 (HER2) are presented in Table 4. As expected in the context of an active breast cancer screening program, most of the participants were of early stage (40.1% stage 1 and 43% stage 2), and of the luminal A subtype (72.4%); 83.7% were positive for hormone receptors. A multivariable linear regression adjusted for estrogen exposure, BMI, and bisphosphonate use in the previous two years found no correlations of baseline DXA BMD (g/cm^2^), *T* score, or *Z* score in any of the three sites examined, with various parameters of breast cancer including: tumor size and grade, hormone-receptor status, nodal involvement, and disease stage.

**Table 3:** Baseline characteristics of all the women with breast cancer included in the study.

Group	Breast cancer *n* = 344
*Age*	
Mean ± SD	58.0 ± 11.6
Median (Q1,Q3)	60 (50,65)
Range	25–85
BMI mean ± SD	28.6 ± 5.4
Postmenopausal HRT use *n* (%)	95 (27.6%)
*Menstrual cycle status* *n* (%)	
Premenopausal	78 (22.7%)
Perimenopausal	9 (2.6%)
Menopause	257 (74.7%)
Parity *median* (Q1,Q3)	3 (2,4)
*Past breastfeeding* *n* (%)	
Never breastfed	44 (12.8%)
After some of the deliveries	63 (18.4%)
After every delivery	236 (68.8%)
*Ethnicity* *n (%)*	
Ashkenazi Jew (one parent or both)	184 (53.5%)
Sephardic Jew (both parents)	128 (37.2%)
Bedouin Arab	10 (2.9%)
Other	22 (6.4%)
*Smoking status* *n* (%)	
Never smoked	231 (67.2%)
Past smoker	64 (18.6%)
Current smoker	49 (14.2%)
Prior fracture of any kind *n* (%)	84 (24.4%)
*Lab results at enrollment*^a^	
Creatinine (mg/dL) mean ± SD	0.69 ± 0.2
eGFR (MDRD calc; mL/min/1.73 m²) mean ± SD	99.8 ± 24.6
PTH (pg/mL) mean ± SD	54.2 ± 28.9
(OH)25-Vitamin D (nmol/L) mean ± SD	47.2 ± 19.2
(OH)25-Vitamin D < 50(nmol/L) *n (%)*	185 (58%)
Duration of unopposed estrogen exposure (years) mean ± SD	14.3 ± 4.4

*BMI* body mass index, *HRT* hormone replacement therapy, *eGFR* estimated glomerular filtration rate, *PTH* parathyroid hormone, *MDRD calc* modification of diet in renal disease calculation.

^a^Valid creatinine and eGFR test results, taken within ±90 days of enrollment, were available for 341 of cases. PTH—311 cases. Vitamin D—319 cases.

**Table 4:** Disease characteristics for all the women with breast cancer.

Group	Breast cancer *n* = 344
Histological subtype *n (%)*	
IDC	303 (88.1%)
ILC	36 (10.5%)
Other	5 (1.5%)
DCIS present *n* (%)	80 (23.3%)
*Tumor-Node-Metastasis (TNM) staging*	
T *n* (%)	
1	194 (56.4%)
2	115 (33.4%)
3	23 (6.7%)
4	6 (1.7%)
N/A	6 (1.7%)
*N* *n (%)*	
0	199 (57.8%)
1	116 (33.7%)
2	19 (5.5%)
3	6 (1.7%)
N/A	4 (1.2%)
*M* *n (%)*	
0	328 (95.3%)
1	16 (4.7%)
*Overall pathological stage*	
Stage *n (%)*	
1	138 (40.1%)
2	148 (43.0%)
3	38 (11.0%)
4	16 (4.7%)
*Histological grade* *n (%)*	
Low	89 (25.9%)
Intermediate	115 (33.4%)
High	59 (17.2%)
N/A	81 (23.5%)
*Immunohistochemistry* *n (%)*	
ER+	284 (82.6%)
PR+	230 (66.9%)
*HER2 overexpression*	
0/1+	226 (65.7%)
2 + Fish**−**	49 (14.2%)
2 + Fish unknown	11 (3.2%)
3+/Fish + (HER2 pos)	58 (16.9%)
*Immunohistochemistry based subgrouping*^a^ *n (%)*	
Luminal A: ER+/PR + & HER2 neg	249 (72.4%)
Luminal B: ER+/PR + & HER2 pos	39 (11.3%)
HER2 pos ER− PR−	19 (5.5%)
Triple negative	37 (10.8%)

*N/A* not available, *DCIS* ductal carcinoma in situ, *IDC* intraductal carcinoma, *ILC* Intralobular carcinoma.

^a^Patients with HER2 2+ and unknown CISH/FISH status were regarded as HER2negative.

### The association of baseline BMD with breast cancer survival

The median follow-up of breast cancer patients was 51.9 (41.1, 62.3) months. As of June 30, 2018, 25 breast cancer patients had died. A Cox regression analysis found no association of DXA BMD, or *T*- and *Z*-scores (with and without adjustment for age and breast cancer stage), with mortality.

## Discussion

This prospective case-control trial confirmed the association we found between higher BMD and breast cancer risk in our retrospective analysis^17^. The current study showed a higher mean femoral neck DXA *Z* score and total hip BMD, and also higher total hip *Z* and *T* scores among women with breast cancer than among matched controls without breast cancer. These findings were despite a significantly lower mean vitamin D level among those with breast cancer. Since the women with breast cancer were matched to the control group according to prior HRT use, our study suggests that bone integrity and breast cancer risk might be linked by factors not related to estrogen. Spine DXA measurements were not different between the breast cancer and control groups. Among the women with breast cancer, DXA BMD was not found to be associated with breast cancer characteristics at diagnosis including tumor size or grade, nodal involvement, disease stage, or hormone receptor status. Nor was DXA BMD associated with mortality among the women with breast cancer.

Our results corroborate several studies that reported higher incidence of breast cancer in postmenopausal woman with higher BMD^2,6–14,18,20–22^. A meta-analysis was conducted of 70,878 postmenopausal women from eight prospective cohort and two nested-control studies. For women in the highest vs. the lowest category of hip and spine BMD, the relative risks of developing breast cancer were 1.66 and 1.82, respectively^15^. The current report of DXA BMD results in women with incident breast cancer compared to matched controls concurs with observations on bone mass in a matched pair analysis of 242 women with newly diagnosed breast cancer in the Marburg breast cancer and osteoporosis trial (MABOT) study^13^. There, bone density, as assessed with quantitative ultrasonometry (QUS) at the heel, revealed higher speed of sound and stiffness index *T*- and *Z*-scores in women with incident breast cancer than in healthy controls, even after post-matching for possible confounding variables of estrogen exposure. In the MABOT II trial, the same group of researchers studied 402 postmenopausal and 88 premenopausal women with breast cancer, and matched controls, using QUS and DXA BMD. Similar to the initial report, significantly higher values were found in all DXA BMD and QUS parameters of postmenopausal women, but not in premenopausal with breast cancer, compared to controls^14^. A similar observation was apparent in our study population; higher BMD was found in BC patients above age 50 compared to controls but not in the younger population. In the MABOT II trial BMD was higher at all three sites (femoral neck, total hip, and spine) while in our study higher BMD in BC patients was found only in total hip BMD, *T* and *Z* scores and femoral neck *Z* score, but not in the spine, although there was a trend for higher BMD *T* score and *Z* score in the spine that did not reach statistical significance. These differences may be explained by the larger size of the MABOT II trial, since in our study spine BMD tended to be higher in BC but this did not reach statistical significance. If our findings are substantiated by other research then possibly a study design using HRpQCT that can evaluate for cortical thickness (in the arm and the tibia) could examine this question in cases and controls, but this is beyond the scope of the study.

Our findings and those of the MABOT studies contrast with a case control study that found non-significantly different BMD at all sites (lumbar spine, hip, radius, and whole body) between 79 postmenopausal women aged 65 years and older, who had been recently diagnosed with breast cancer, and 158 age-matched controls with normal mammograms^19^. Both our cohort and that of the MABOT studies assessed younger women, mean age 57–59 years. This may explain the discrepancy between observations, as additional parameters may affect BMD in older age women, and thus confound the association between BMD and incident breast cancer.

Lifetime unopposed estrogen exposure may have confounded our findings, such that longer exposure may have led to better BMD while increasing the risk for breast cancer. However, since we did not collect relevant data for calculating this parameter in the control group, we cannot draw conclusions regarding higher BMD as a risk factor for breast cancer, independent of lifetime estrogen exposure. Notably, in the large MABOT II study, the higher BMD in the breast cancer group could not be explained by differences in years of lifetime estrogen exposure, age at menarche, or duration of HRT use^14^.

We did not find associations of DXA BMD with breast cancer characteristics at diagnosis, including tumor size or grade, nodal involvement, disease stage, hormone receptor status, or survival. Notably, during 6.5 years of follow up of a large cohort of elderly women (mean age 70.6 years) who performed single-photon absorptiometry BMD, those with high BMD had an increased risk especially for advanced breast cancer (stage II or higher)^30^. Moreover, a prospective study of 1504 women aged 75 years and older reported a greater risk of death during 7 years follow up for those in the highest than the lowest tertile at all skeletal sites (trochanter, Wards’ triangle, and femoral neck)^31^. The younger age and the shorter length of follow up of the women with breast cancer in our cohort may partially explain the discrepancy in findings.

We report higher total hip BMD among women with breast cancer compared to a control group, despite lower mean vitamin D levels. In multiple studies and meta-analyses, low vitamin D levels were found to be associated with increased risk of breast cancer^24,25^. Taken together, these findings imply an independent association of BMD with breast cancer risk, which is not mediated by vitamin D levels.

A main strength of this study is the unique structure of the breast cancer clinic in our institution, which serves a diverse and large population. This made patient recruitment more feasible, and thus enabled adequate case control matching and uniform blood testing. Since all the breast cancer patients were recruited in a single center, thorough characterization of disease features was possible for all the participants. The prospective nature of our study and the use of DXA BMD upon the diagnosis of breast cancer are also unique to our study. In contrast, most studies stratified the future risk of developing breast cancer according to DXA BMD group. Our in-house mammography and BMD units enabled greater standardization of these tests. Another strength of our study is that we matched women with breast cancer to a control group according to postmenopausal HRT use. This mitigates the possibility that the higher BMD in the breast cancer group resulted from greater use of HRT, and suggests that bone integrity and breast cancer risk might be linked by factors other than estrogen effect.

Our study has several limitations. We did not exclude women who were treated for osteoporosis during or within the two years prior to study entry, and this may have affected BMD. We addressed this issue after the study ended and found low rates of prior bisphosphonate treatment, which were similar in the breast cancer and control groups. We excluded patients who received neoadjuvant chemotherapy (NAC), used primarily during years of the study to enable breast conserving surgery, and therefore may have skewed our cohort to earlier stage disease with a better prognosis and a higher percentage of HR positive cases. Standard-of-care NAC is currently administered not only to facilitate breast conservation therapy but also to patients with HER2 positive or triple negative subtypes of breast cancer with tumors 2 cm or larger in order to individualize post-surgical therapy on the basis of pathologic response to NAC^32^. Investigation of BMD in patients receiving NAC is warranted. The ethnicity of our population was over 90% Jewish, of either European (Ashkenazi) or North-African/Asian (Sepharadi) origins. The findings in our population are probably relevant for European and American populations as shown by the similarity of the order of prevalence of the different types of cancers in the USA and Israel^33,34^. In addition, we were unable to calculate lifetime estrogen exposure in the control group, and therefore the possibility of estrogen exposure as a confounder could not be assessed. Exercise and physical activity level may impact BMD and potentially breast cancer risk but unfortunately, we did not collect this demographic information in BC and controls. Finally, our sample size did not enable stratifying by menopausal status at breast cancer diagnosis; this may have affected our results.

In conclusion, our prospective case-control study showed higher femoral neck DXA *Z* scores, and total hip BMD, *Z*, and *T* scores, among women diagnosed with breast cancer than among matched controls without breast cancer, despite significantly lower mean vitamin D levels in the former. DXA BMD parameters were not found to be associated with breast cancer characteristics at diagnosis. In addition, DXA BMD was not associated with mortality among women with breast cancer.

## Methods

The study was conducted at Soroka University Medical Center, and approved by the institutional review board: IRB # SOR11-189. Prior to participation, women with breast cancer and women in the healthy control group signed informed consent forms.

### Population

Israel has operated a comprehensive breast cancer early-detection program since the early 1990s, in the context of comprehensive universal national health care coverage^35^. Soroka University Medical Center is a regional medical center for a population of ~700,000, and serves nearly 1.2 million as a tertiary hospital. This includes the provision of mammographic screening and breast cancer treatment.

Inclusion criteria for the study group were age 18 years and older and a diagnosis of breast cancer. Women were recruited at their first visit to the oncology department following surgery for breast cancer. Radiotherapy and chemotherapy prior to screening were exclusion criteria. The control group was recruited at the mammography unit following a breast mammography or breast ultrasound that was reported as normal. Exclusion criteria for the study and control groups were oncologic diseases other than breast cancer, bone diseases other than osteoporosis, autoimmune diseases (i.e. rheumatoid arthritis and systemic lupus erythematosus), and chronic steroid therapy.

Women in the breast cancer and control groups were matched by age ±5 years, BMI ± 5 kg/m^2^, parity ±1 birth, and the use of postmenopausal hormone replacement therapy (HRT).

### Primary objective

The primary objective of this study was to compare BMD between women newly diagnosed with breast cancer and matched healthy women with no radiologic evidence of breast cancer.

### Study procedures

During the first visit, the following data were recorded: body weight and height, demographic data, and past medical history. The latter included postmenopausal HRT, smoking habits, menstrual cycle status, parity and past breastfeeding. Blood samples were taken for measurement of vitamin D levels, parathyroid hormone (PTH), thyroid stimulating hormone (TSH), creatinine, calcium, phosphorous, albumin, complete blood count, and C-reactive protein. Estimated glomerular filtration rate (eGFR) was calculated by the modification of diet in renal disease (MDRD) GFR equation: 186 × serum creatinine^−1.154^ × age^−0.203^ × 0.742.

### BMD measurement

BMD was performed one year before or up to 3 months after enrollment for the breast cancer group, and one year before or after enrollment for the control group. BMD was measured by DXA using a Prodigy densitometer (GE-Lunar, Milwaukee, USA).

BMD was assessed at the lumbar spine, femoral neck, and total hip; and expressed as bone density (in g/cm^2^), *T*-score (standard deviation from the mean for young women), and *Z*-score (standard deviation from the mean for age-matched women adjusted for body mass). *Z* and *T* scores were calculated according to the Lunar-Prodigy manufacturer’s internal database, and based on data of The National Health and Nutrition Examination Survey III.

### Breast cancer data

From the institutional medical records, the following breast cancer data were extracted: histological subtype; the presence of ductal carcinoma in situ in the specimen; tumor grade; IHC staining for ER, PR, and the expression of HER2; TNM staging; and the type of primary surgery. All the women with breast cancer underwent tissue diagnosis and immunostaining. For most of those with equivocal (+2) HER2 immunostaining, tumor tissues were submitted for chromosome in situ hybridization or fluorescent in situ hybridization testing. Participants were classified into four subtypes by IHC staining results: luminal A- ER and/or PR positive and HER2 negative; luminal B- ER and/or PR positive and HER2 positive; HER 2 positive- ER negative/PR negative/HER2 positive; triple negative- ER negative/PR negative/HER2 negative. (Women with HER2 2+ results and unknown chromosome in situ hybridization/fluorescence in situ hybridization status were regarded as HER2 negative).

### 25 OH vitamin D assay

Vitamin D status was reported when available. Vitamin D levels were determined by measuring patients’ serum 25-hydroxyvitamin D (25(OH)D) levels by chemiluminescent immunoassay technology, LIAISON^®^ 25 OH Vitamin D TOTAL Assay ([REF] 310600). The results were expressed as nmol/l, normal range: 75–250 nmol/L.

### PTH assay

PTH status was reported when available. PTH was measured using the intact-PTH test [Immulite 2000 and ADVIA Centaur, Siemens]. This test is based on a chemiluminescent reaction in two sites on the molecule. PTH levels were reported in pg/mL; normal range: 14–72 pg/mL.

### Drug distribution data

Anti-osteoporotic medications used in the two years prior to enrollment were identified by the Anatomical Therapeutic Chemical Classification System codes and collected from the electronic medical record, which were accessible for participants insured by Clalit Health Services. Medications included oral bisphosphonates (ATC codes M05BA04 and M05BA07), injected bisphosphonates (M05BA03 and M05BA08), vitamin D analogs (A11CB and A12AX), and calcium supplements (A02AC, A12AA, and A12AX).

### Lifetime estrogen exposure

Lifetime estrogen exposure was calculated only for the breast cancer group, and was based on self-reported data, using the following equation:

Total menstrual period/2 [total menstrual duration = menopause age–(menarche age + parity*9 months + miscarriage*3 months + breastfeeding duration + pill use duration)]^36^.

### Statistical analysis

Categorical data were expressed as numbers and percentages. Continuous data were expressed as means ± standard deviations or medians (Q1, Q3), depending on the normality of the data. Parametric model assumptions were assessed using normal plot. For categorical data, univariable comparisons were made using *χ*^2^-test or Fisher’s exact test, as appropriate. For continuous variables, the independent sample *T*-test or the Mann–Whitney *U* test was applied, as appropriate. Pearson’s correlation was used to assess bivariate relations. To further examine associations of BMD with tumor size, tumor grade, nodal involvement, and breast cancer stage, we performed multivariable linear regression models, adjusted for estrogen exposure, BMI, and bisphosphonates received in the previous 2 years. Variables were tested for multicollinearity. Survival analysis by DXA results was done using Cox regression models, while adjusting for age and breast cancer stage. All *p* values are based on 2-tailed tests of significance. Variables were considered significant at *p* < 0.05. Statistical analysis was performed with IBM SPSS version 24.

### Reporting summary

Further information on research design is available in the Nature Research Reporting Summary linked to this article.

## Supplementary information

Reporting Summary Checklist

## Data Availability

Due to the national regulations anonymized data can be shared after approval of the local IRB and data sharing committee. The interested researchers should contact the corresponding author.
